# Elevated microRNA-129-5p level ameliorates neuroinflammation and blood-spinal cord barrier damage after ischemia-reperfusion by inhibiting HMGB1 and the TLR3-cytokine pathway

**DOI:** 10.1186/s12974-017-0977-4

**Published:** 2017-10-23

**Authors:** Xiao-Qian Li, Feng-Shou Chen, Wen-Fei Tan, Bo Fang, Zai-Li Zhang, Hong Ma

**Affiliations:** Department of Anesthesiology, First Affiliated Hospital, China Medical University, Shenyang, Liaoning 110001 China

**Keywords:** Blood-spinal cord barrier, High-mobility group box-1, Ischemia-reperfusion injury, MicroRNAs, Toll-like receptor 3

## Abstract

**Background:**

Ischemia-reperfusion (IR) affects microRNA (miR) expression and causes substantial inflammation. Multiple roles of the tumor suppressor miR-129-5p in cerebral IR have recently been reported, but its functions in the spinal cord are unclear. Here, we investigated the role of miR-129-5p after spinal cord IR, particularly in regulating high-mobility group box-1 (HMGB1) and the Toll-like receptor (TLR)-3 pathway.

**Methods:**

Ischemia was induced via 5-min occlusion of the aortic arch. The relationship between miR-129-5p and HMGB1 was elucidated via RT-PCR, western blotting, and luciferase assays. The cellular distribution of HMGB1 was determined via double immunofluorescence. The effect of miR-129-5p on the expression of HMGB1, TLR3, and downstream cytokines was evaluated using synthetic miRs, rHMGB1, and the TLR3 agonist Poly(I:C). Blood-spinal cord barrier (BSCB) permeability was examined by measuring Evans blue (EB) dye extravasation and the water content.

**Results:**

The temporal miR-129-5p and HMGB1 expression profiles and luciferase assay results indicated that miR-129-5p targeted HMGB1. Compared with the Sham group, the IR group had higher HMGB1 immunoreactivity, which was primarily distributed in neurons and microglia. Intrathecal injection of the miR-129-5p mimic significantly decreased the HMGB1, TLR3, interleukin (IL)-1β and tumor necrosis factor (TNF)-α levels and the double-labeled cell count 48 h post-surgery, whereas rHMGB1 and Poly(I:C) reversed these effects. Injection of miR-129-5p mimic preserved motor function and prevented BSCB leakage based on increased Basso Mouse Scale scores and decreased EB extravasation and water content, whereas injection rHMGB1 and Poly(I:C) aggravated these injuries.

**Conclusions:**

Increasing miR-129-5p levels protect against IR by ameliorating inflammation-induced neuronal and BCSB damage by inhibiting HMGB1 and TLR3-associated cytokines.

## Background

Spinal cord ischemia-reperfusion (IR) injury is a severe but unpredictable and unpreventable complication that commonly occurs during thoracoabdominal aortic surgery. IR-induced inflammatory responses, including increases in extracellular matrix degradation and vascular permeability, can aggravate spinal cord edema and worsen neurologic deficits, resulting in a high incidence of paraplegia [1, 2]. Treatments that might regulate the above mechanisms have become a focus in the clinic [3, 4]. Previous studies have shown that increases in microglia and upregulation of Toll-like receptors (TLRs) are associated with proinflammatory cytokine release through recognition of damage-associated molecular pattern molecules (DAMPs) during spinal cord ischemia [4, 5]. High-mobility group box-1 (HMGB1) is a well-characterized DAMP that is conserved in most mammals [6]. Under physiological conditions, HMGB1 functions to stabilize nucleosomes. In various models of injury, HMGB1 is immediately released by injured cells to establish and amplify inflammatory responses via receptor for advanced glycation end products (RAGE) or TLRs through the release of chemokines and cytokines [7–10]. We have demonstrated that activation of the TLR4-mediated nuclear factor-kappa B (NF-κB)/interleukin (IL)-1β positive feedback loop promotes substantial inflammatory damage to neurons and the blood-spinal cord barrier (BSCB) after spinal cord IR [5]. Moreover, the hyper-secreted HMGB1 is detected before the secretion of tumor necrosis factor (TNF)-α and IL-1β during spinal cord injury [7]. Therefore, we hypothesize that HMGB1 is an upstream modulator of the TLR-mediated pathway in the spinal cord. Treatments that regulate the interactions between TLRs and HMGB1 may be used as novel therapeutic approaches for spinal cord IR injury.

MicroRNAs (miRs) are 20–22-nucleotide-long non-coding RNAs that are specifically expressed in certain organs or cells to negatively regulate target gene expression at the posttranscriptional level [11, 12]. Many miRs have been shown to be dysregulated during central nervous system (CNS) injury [11, 12]. Recently, miR-129-5p, which is known to function as a tumor suppressor [13], has been shown to function in neuro-apoptosis by regulating bcl-2 and caspase-3 expression during cerebral IR [11]. miR-129-5p has also been shown to modulate angiogenesis and revascularization by regulating vascular endothelial growth factor expression via RAGE-HMGB1 signaling during intracerebral hemorrhage [14]. Additionally, miR-129-5p prevents NF-κB transduction and subsequent inflammatory infiltration in autoimmune diseases by inhibiting TLR2 or TLR4-HMGB1 signaling [10, 15]. TLR3, which was originally identified as the receptor for viral RNA, has been demonstrated to be essential for HMGB1 in facilitating agonists with other TLRs during neuroinflammation and neurodegeneration [16, 17]. Upregulation of TLR3 stimulates NF-κB transcription and increases the expression of neurodegeneration markers [16]. In contrast, TLR3-deficient mice are resistant to galactosamine sensitization and display reduced production of inflammatory cytokines [18]. However, little is known about the function of miR-129-5p in the interaction between HMGB1 and TLR3 in mouse models of spinal cord IR. In this study, first, we investigated whether miR-129-5p was dysregulated and then defined HMGB1 as its target in this context. Next, we assessed the effects of miR-129-5p on HMGB1 expression and the TLR3-cytokine pathway in vivo by intrathecal injection of an miR mimic and control, recombinant HMGB1 (rHMGB1), and a TLR3-specific agonist (polyinosinic-polycytidylic acid, Poly(I:C)). Additionally, the effects of these treatments on hind-limb motor function and BSCB leakage were evaluated to determine the role of miR-129-5p in spinal cord IR injury.

## Methods

### Animals

The 12- to 15-week-old C57BL6 mice used in this study were purchased from the Animal Center of China Medical University (Shenyang, China). All experiments were approved by the Ethics Committee of China Medical University and were performed in accordance with the Guide for the Care and Use of Laboratory Animals (U.S. National Institutes of Health publication no. 85-23, National Academy Press, Washington, DC, USA). The mice were given free access to food and water and were allowed to acclimatize in a standard cage for a minimum of 7 days at 22–24 °C with a 12-h light/dark cycle before surgery.

### Mouse model of spinal cord IR injury

The mouse IR model was designed as previously reported [4]. Briefly, the aortic arch was exposed through a cervicothoracic incision and cross-clamped between the left common carotid artery and the left subclavian artery for 5 min to induce ischemia. A 90% decrease in the blood flow in the tail artery was confirmed with a Doppler monitor (Moor Instruments, Axminster, UK). Then, the clamps were removed to induce reperfusion for 48 h. The sham-operated mice underwent the same procedure without occlusion.

### Intrathecal injection of a synthetic miR-129-5p mimic and a control

Intrathecal injections were performed as previously described [19]. Briefly, after anesthetization with sevoflurane, a 10-μL microsyringe (Gaoge Co., Ltd., Shanghai, China) was inserted between segments L_5–6_ of the dura in the presence of a tail flick. Intrathecal injections were repeatedly performed for 3 days prior to ischemia. According to the manufacturer’s guidelines, we intrathecally infused 10 μL of a synthetic miR-129-5p mimic (mimic-129) and a control mimic (con-129) (Jima Inc., Shanghai, China) with Lipofectamine 2000 (Invitrogen, MA, USA) to modulate in vivo miR-129-5p expression. The sequences of mimic-129 and con-129 are 5′-CUUUUUGCGGUCUGGGCUUGC-3′, 5′-AAGCCCAGACCGCAAAAAGUU-3′ and 5′-UUCUCCGAACGUGUCACGUTT-3, 5′-ACGUGACACGUUCGGAGAATT-3′, respectively.

### Experimental protocol

**Fig. 1:**
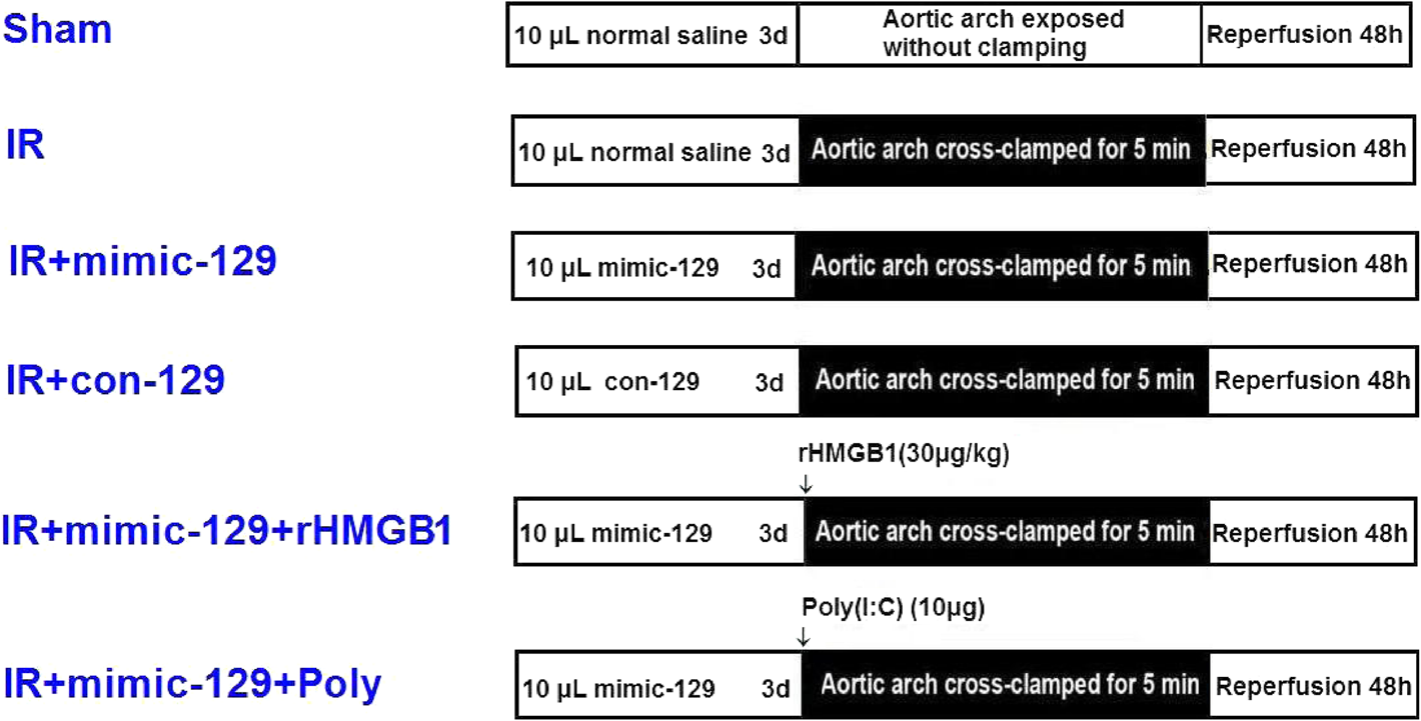
Experimental groups and protocol. Schematic representation of the six groups of mice exposed to the different treatments

Mice were randomly assigned to one of the six groups as shown in Fig. 1 and as follows: (1) Sham group: mice subjected to the surgical procedure without ischemia; (2) IR group: mice given an intrathecal injection of 10 μL of normal saline; (3) IR + mimic-129 group (mimic-129 group): mice given an intrathecal injection of 10 μL of mimic-129 (40 μM); (4) IR + con-129 group (con-129 group): mice given an intrathecal injection of 10 μL of con-129 (40 μM); (5) IR + mimic-129 + rHMGB1 group (rHMB1 group): mice from the mimic-129 group given 10 μL of rHMGB1 (30 μg/kg, SinoBio, Shanghai, China) immediately prior to ischemia; and (6) IR + mimic-129 + Poly(I:C) group (Poly group): mice from the mimic-129 group given 10 μL of Poly(I:C) (1 μg/μL, Invivogen, USA) immediately prior to ischemia. The mice were euthanized 12 and 48 h after surgery with an overdose of sevoflurane. Spinal cord segments L_4–6_ were collected for analysis.

### Luciferase assay

Potential binding between miRNA-129-5p and the 3′ untranslated region (3′UTR) of HMGB1 (NM_010439) was predicted using TargetScan (http://www.targetscan.org/). Using Lipofectamine 3000, the cells were co-transfected with mimic-129, con-129, and a luciferase reporter vector containing the wild-type (WT) 3′UTR (5′-TACCACTCTGTAATTGCAAAAAAAAA-3′) or mutant (MT) 3′UTR (5′-GAATACCACTCTGTAATTTACCCCCAAAA-3′). After transfection for 48 h, the luciferase activity was measured with the Dual-Luciferase Reporter Assay Kit (Promega Corp., Madison, WI, USA) according to the manufacturer’s instructions. Renilla luciferase activity was used to normalize the firefly luciferase activity.

### Quantification of the miR and HMGB1 levels

miR-129-5p and HMGB1 expression was measured in triplicate with the Applied Biosystems 7500 Real-Time PCR System (Foster City, CA, USA). Total RNA from the L_4–6_ spinal cord segments was extracted using the TRIzol reagent (Invitrogen, Carlsbad, CA, USA). miR-129-5p was quantified using the TaqMan MicroRNA Assay Kit (Applied Biosystems) [20]. The following primers were used:

miR-129-5p (forward, 5′-ACACTCCTTTTTGCGTCTGGGCTTGC-3′ and reverse, 5′-TGGTGTCGTGGAGTCG-3′) and HMGB1 (forward, 5′-GGAGTGGCTTTTGTCCCTCAT-3′ and reverse, 5′-TGCCTCTCGGCTTTTTAGGA-3′). The relative expression levels were normalized to U6 (forward, 5′-CTCGCTTCGGCAGCACA-3′ and reverse, 5′-AACGCTTCACGAATTTGCGT-3′) and GAPDH (forward, 5′-GGTTGTCTCCTGCGACTTCA-3′ and reverse, 5′-GGTGGTCCAGGGTTTCTTACT-3′). The results were analyzed with the 2^−ΔΔCt^ method.

### Western blotting

Spinal proteins were collected and purified with a protein extraction kit according to the manufacturer’s instructions (KC-415, KangChen, Shanghai, China). After electrophoresis and transfer, the proteins were incubated overnight at 4 °C with primary antibodies against HMGB1 (MyBioSource, CA, USA) and TLR3 (Abcam, Cambridge, MA, USA). GAPDH (Abcam, Cambridge, MA, USA) was used as the control. The protein bands were visualized with an ECL kit (Biyuntian, Beijing, China) and quantified using the Quantity One software (Bio-Rad Laboratories, Milan, Italy).

### Double immunofluorescence analysis

Double immunofluorescence analysis was performed to detect HMGB1 and a neuronal marker (NeuN), a microglial marker (Iba1), or an astrocytic marker (GFAP) as previously described [2]. Briefly, 30-μm-thick sections were incubated overnight at 4 °C with the primary mouse anti-HMGB1 (MyBioSource, CA, USA) and rabbit anti-NeuN, rabbit anti-Iba1, or rabbit anti-GFAP (Abcam, Cambridge, MA, USA) antibodies. Then, the sections were incubated with Alexa 488-conjugated donkey anti-rabbit IgG and Alexa 594-conjugated donkey anti-mouse IgG (Molecular Probes, MA, USA) for 2 h at room temperature. Additionally, the interaction between HMGB1 and TLR3 was explored using primary rabbit anti-HMGB1 and mouse anti-TLR3 antibodies as described above. Images were captured using a Leica confocal microscope (Leica Microsystems, Buffalo Grove, IL, USA).

### Behavioral assessment

The hind-limb motor function was scored on a Basso Mouse Scale for locomotion from 0 (complete paraplegia) to 9 (normal gait) [4]. Function was assessed every 12 h during a 48-h observation period by two investigators who were blinded to the experimental procedures.

### Blood-spinal brain barrier permeability assessment

BSCB permeability was detected by Evans blue (EB) extravasation and the spinal water content [2]. EB (20 mg/kg, Sigma, MO, USA) was slowly administered through the tail vein 60 min before sacrifice. The tissues were cut into 10-μm sections and visualized with a BX-60 fluorescence microscope (Olympus, NY, USA) with a green filter. For the quantitative evaluation, the absorption of the supernatant was detected at 632 nm and reported relative to the wet tissue weight (μg/g) according to a standard curve.

The spinal water content was measured using the wet-dry method and calculated as (wet weight − dry weight) × 100/wet weight [2, 3].

### Measurement of IL-1β and TNF-α using ELISAs

The IL-1β and TNF-α concentrations were determined with ELISA kits (R&D Systems, Minneapolis, MN, USA) according to the manufacturer’s instructions. Each sample concentration was calculated based on the standard curve.

### Statistical analysis

The data are presented as the mean ± standard deviation (SD) and were compared using the *t* test or one-way analysis of variance (ANOVA) followed by post hoc analysis with the SPSS software (version 19.0, SPSS Inc., Chicago, IL, USA). *P* < 0.05 was considered significant.

## Results

### Temporal expression of miR-129-5p and HMGB1 after IR

**Fig. 2:**
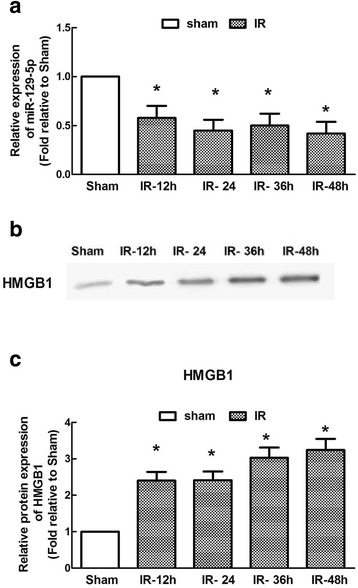
Time course of IR-induced alterations in the miR-129-5p and HMGB1 levels in a mouse model of spinal cord IR injury. **a** Quantification of miR-129-5p expression. **b** Representative western blots to detect HMGB1 expression. **c** Quantification of the integrated intensity of the HMGB1 bands. Data are expressed as the mean ± SD. *n* = 6 per group. *P < 0.05 versus the Sham group

The IR-induced changes in the miR-129-5p and HMGB1 expression levels were examined at 12-h intervals for 48 h post-surgery. miR-129-5p expression was obviously downregulated with time and reached its lowest levels at both 12 and 48 h after IR compared with the levels in the sham surgery group (Fig. 2a, *P* < 0.05). Likewise, the HMGB1 protein levels were significantly increased beginning from 12 h after IR, and this high level was maintained throughout the observation period (Fig. 2b, c, *P* < 0.05), suggesting a potential negative correlation between miR-129-5p and HMGB1 expression.

### HMGB1 as a target of miR-129-5p

**Fig. 3:**
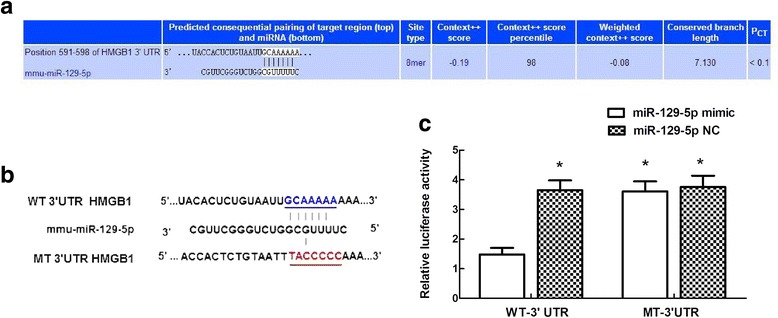
Confirmation of a direct interaction between miR-129-5p and HMGB1. **a** Schematic representation of the predicted interaction between miR-129-5p and HMGB1 based on TargetScan. **b** Binding site of miR-129-5p in the WT and MT 3′UTR of HMGB1. **c** Dual-luciferase reporter gene assay. Luciferase activity was measured after 48 h of co-transfection of HEK-293 cells with the miR-129-5p mimic and the WT or MT reporter vector. Compared to the control, miR-129-5p significantly decreased the luciferase activity of the WT vector but not that of the MT vector. Data are expressed as the mean ± SD. *P < 0.05 versus the control miR

Based on the TargetScan database, seven nucleotides in the 3′UTR of HMGB1 were found to be completely complementary to miR-129-5p (Fig. 3a). Additionally, mimic-129 significantly decreased the luciferase activity of the WT 3′UTR of HMGB1 without affecting the MT 3′UTR (Fig. 3c, *P* < 0.05). No differences were detected between the two groups of cells transfected with con-129, suggesting that HMGB1 was a direct target for miR-129-5p (Fig. 3c, *P* > 0.05).

### Cellular distribution of HMGB1 in the spinal cord after IR

**Fig. 4:**
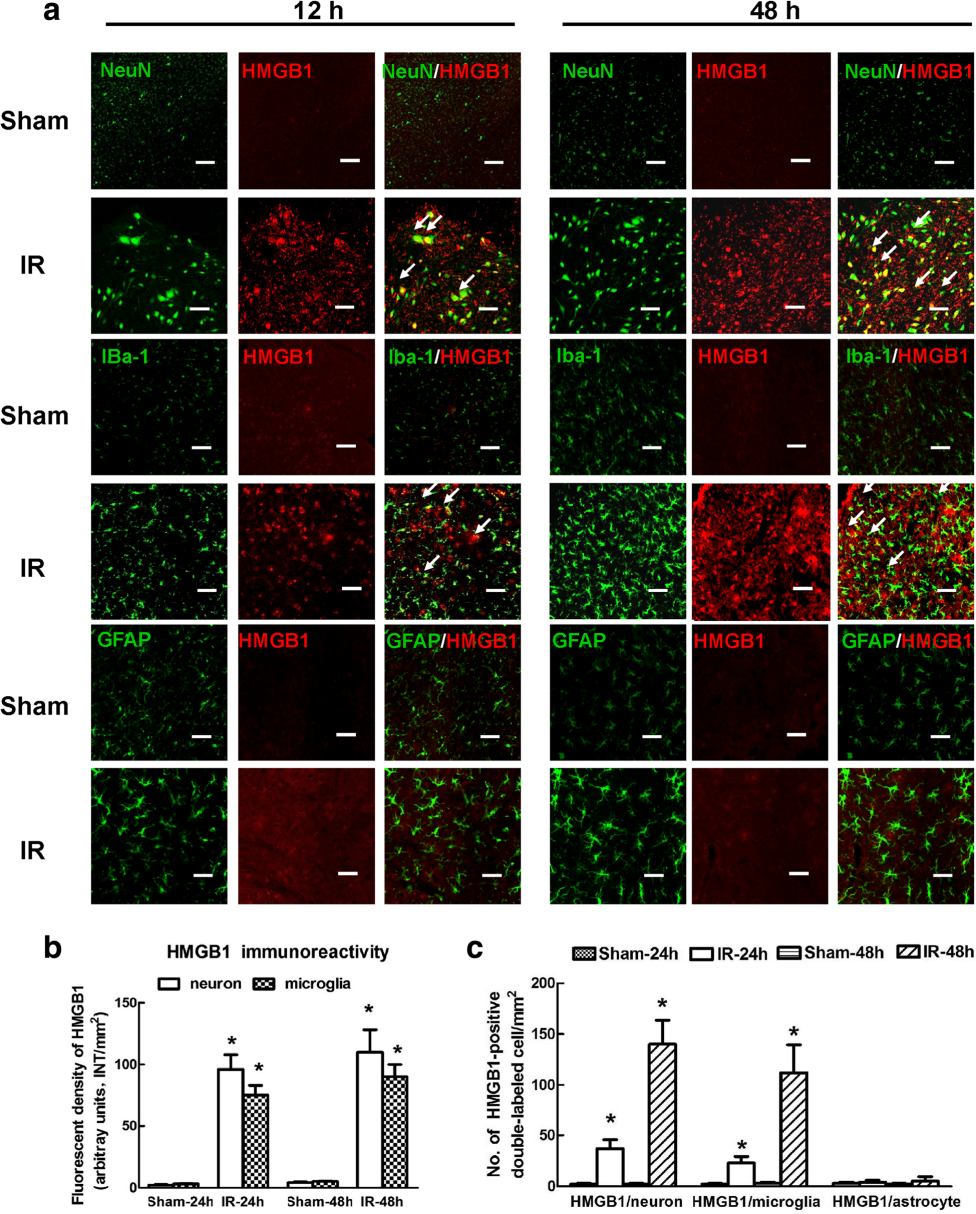
Double immunofluorescence of HMGB1 with markers of major cell types in the spinal cord after IR. **a** Representative fluorescence images of the distribution of HMGB1 (red) in neurons (NeuN; green), microglia (Iba1; green) and astrocytes (GFAP; green) in the spinal cords at 12 and 48 h after IR. Arrows indicate co-localization. Scale bars = 50 μm. **b** Quantification of HMGB1 signals was performed based on the average of three independent images. **c** Quantification of HMGB1-positive neurons and microglia in the spinal cords at 12 h and 48 h after IR. Data are expressed as the mean ± SD. *P < 0.05 versus the Sham group

Double immunofluorescence analysis of HMGB1 and markers of major cell types in the spinal cord was performed at 12 h and 48 h after IR because the lowest levels of miR-129-5p were detected at these time points (Fig. 2). As shown in Fig. 4a, the majority of the fluorescence signal for HMGB1 in the IR group was localized in the cells positive for NeuN and Iba1 (cells with yellow signals) at both 12 and 48 h after IR. This distribution was not observed with the fluorescence signal for GFAP during any of the time points (Fig. 4c, *P* > 0.05), suggesting the HMGB1 was only upregulated in neurons and microglia but not in astrocytes after IR. Furthermore, HMGB1 expression in the injured spinal cords increased over time, as shown by an increase in the HMGB1 staining intensity and the number of double-labeled cells in the quantitative analysis (Fig. 4b, c *P* < 0.05).

### miR-129-5p mimic prevented the upregulation of HMGB1 and the TLR3-cytokine pathway after IR

**Fig. 5:**
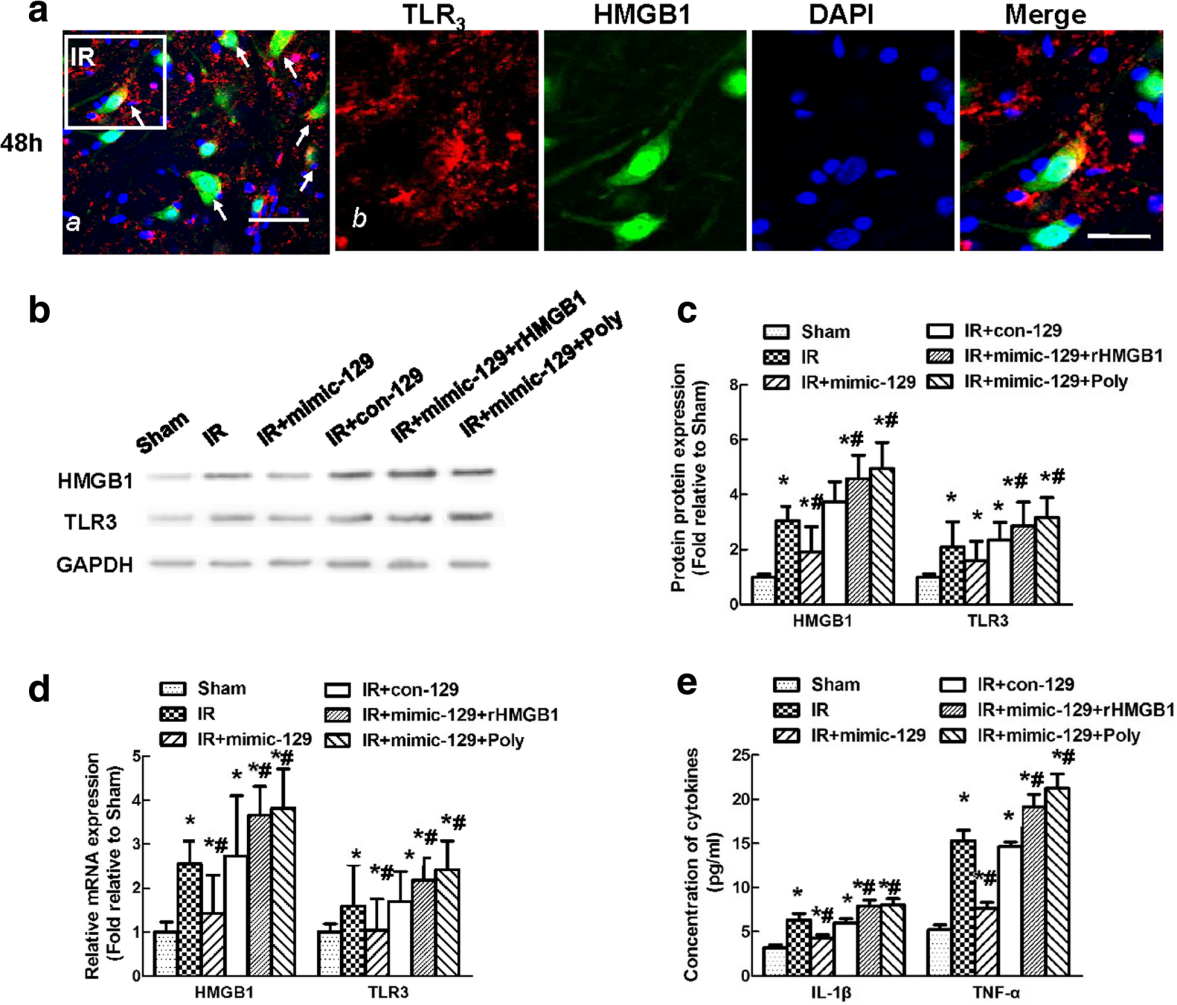
Effect of the miR-129-5p mimic and mimic control on the expression of HMGB1 and TLR3-mediated cytokines at 48 h after IR. **a** Representative fluorescence images of the distribution of TLR3(red), HMGB1(green) and nuclei (blue) in the spinal cords at 48 h after IR. **b** Representative western blots of HMGB1 and TLR3 expression. GAPDH was used as a loading control. **c** Quantification of the relative protein expression of HMGB1 and TLR3 at 48 h after IR. **d** Quantification of the relative mRNA expression of HMGB1 and TLR3 at 48 h after IR. **e** Quantification of spinal IL-1β and TNF-α production at 48 h after IR. All data were obtained from three independent experiments and expressed as the mean ± SD. *P < 0.05 versus the Sham group. ^#^ *P* < 0.05 versus the IR group

**Fig. 6:**
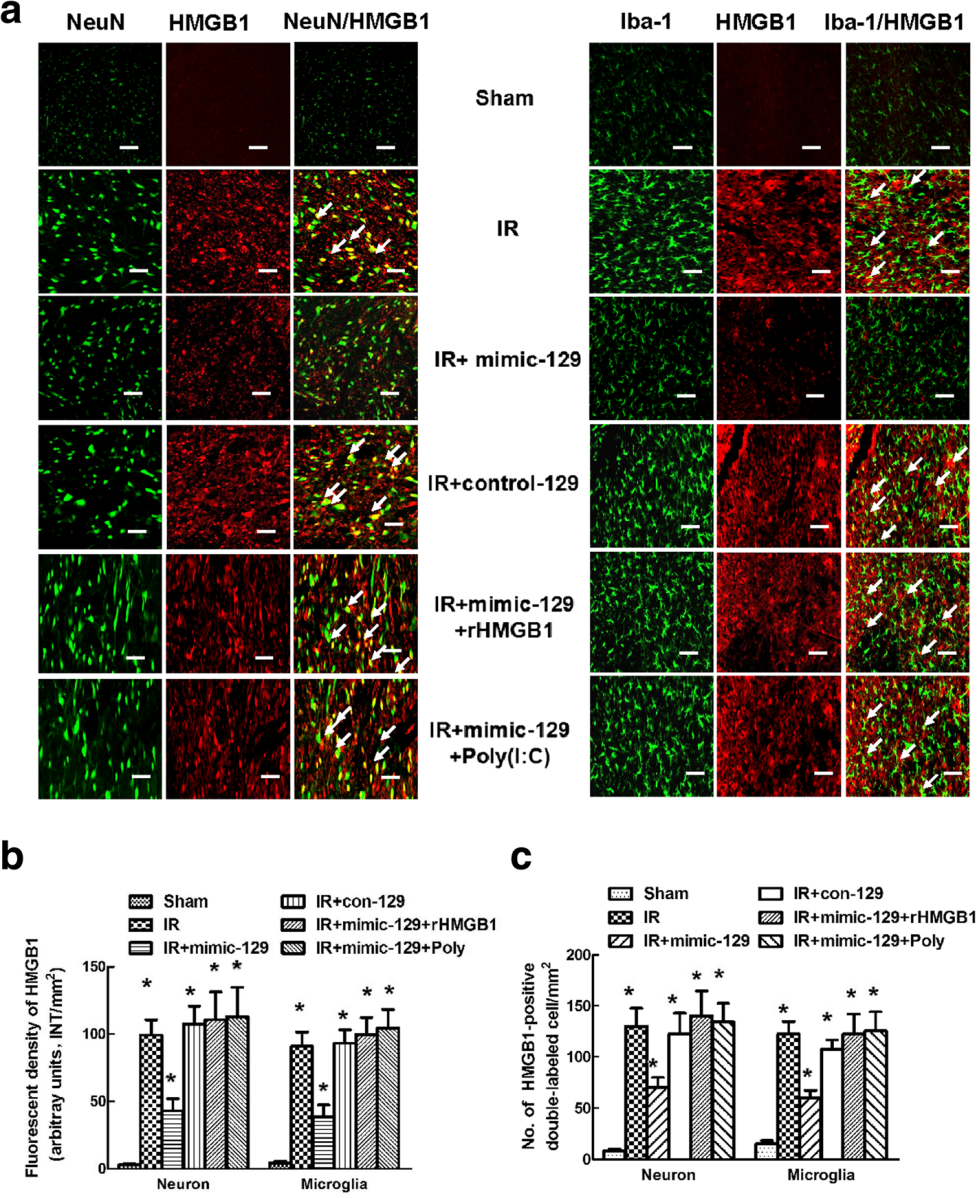
Effects of the miR-129-5p mimic and mimic control on HMGB1 expression in specific cell types of the spinal cord after IR. **a** Representative photomicrographs showing the localization of the fluorescence signals for HMGB1 in neurons and microglia at 48 h after IR. Arrows indicate co-localization. Scale bars = 50 μm. **b** Quantification of HMGB1 signals was performed based on the average of three independent images. **c** Quantification of HMGB1-positive neurons and microglia in the spinal cords at 48 h after IR. Data are expressed as the mean ± SD. *P < 0.05 versus the Sham group. ^#^ *P* < 0.05 versus the IR group

The effects of mimic-129 and con-129 on the regulation of HMGB1 expression were assessed when the HMGB1 immunoreactivity was at its peak. Because TLR3 was located on the cell surface and HMGB1 was distributed in the cytoplasm of the same cells (Fig. 5a), the interaction between HMGB1 and TLR3 or other downstream proinflammatory cytokines was explored via intrathecal injection of rHMGB1 and Poly(I:C) in a series of experiments. Compared with the Sham group, intrathecal injection of mimic-129 significantly inhibited the HMGB1 and TLR3 mRNA and protein levels at 48 h in the IR group, and these effects were abrogated in the presence of rHMGB1 or upregulated TLR3 due to Poly(I:C) (Fig. 5b–d, *P* < 0.05). Representative photomicrographs and quantification showed that mimic-129 injection significantly decreased HMGB1 immunoreactivity and the number of HMGB1-positive double-labeled cells, whereas no such change was observed with con-129 injection. These effects in response to mimic-129 were further increased by the addition of rHGMB1 and Poly(I:C) (Fig. 6b, c, *P* < 0.05). No significant differences were found between the IR and con-129 groups at any of the time points (*P* > 0.05).

Additionally, production of the cytokines IL-1β and TNF-α changed with the same trends observed for HMGB1 and TLR3 production (Fig. 5e, *P* < 0.05). Interestingly, higher HMGB1, TLR3, and associated cytokine levels were observed in response to rHMGB1 and Poly(I:C) injection but not an injection with the synthetic miRs (Fig. 5b–e, *P* < 0.05).

### miR-129-5p mimic improved hind-limb motor function after IR

**Fig. 7:**
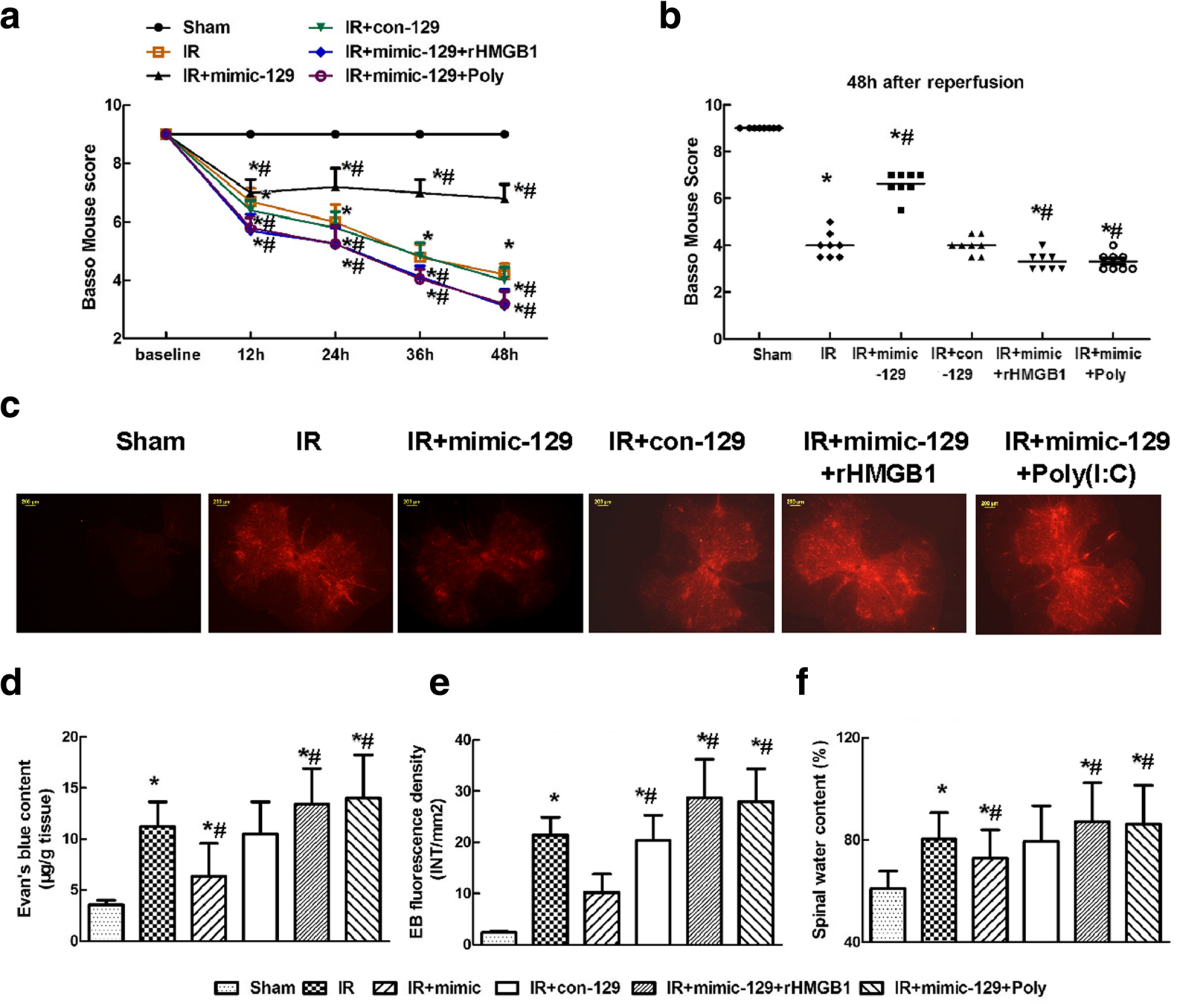
Effects of the miR-129-5p mimic and mimic control on hind-limb motor function and blood-spinal cord barrier (BSCB) integrity after IR. **a** Hind-limb motor function was assessed at 12-h intervals for 48 h after reperfusion using the Basso Mouse Scale for locomotion with a range from 0 (paraplegia) to 9 (normal). **b** Assessment of each mouse at 48 h post-surgery in the six groups. Each symbol represents one mouse. The bar represents the median. **c** Changes in BSCB permeability were measured based on EB extravasation visualized as red fluorescence by microscopy. **d** Quantification of the EB content of the spinal cord (μg/g tissue). **e** Quantification of the EB fluorescence intensity (INT/mm^2^). **f** Quantification of the spinal water content (%). Data are expressed as the mean ± SD. *P < 0.05 versus the Sham group. ^#^ *P* < 0.05 versus the IR group

All mice displayed normal neurological functions before surgery. Ischemia induced by 5-min occlusion of the aortic arch resulted in reduced Basso Mouse Scale scores in all mice at the observed time points (Fig. 7a, *P* < 0.05), indicating motor function deficits. Compared with the untreated mice in the IR group, mice pretreated with mimic-129 had higher average scores (*P* < 0.05), whereas the mice pretreated with con-129 had comparable scores (*P* > 0.05). In contrast, much lower scores were observed in the mice injected with rHMGB1 or Poly(I:C) at the above time points (*P* < 0.05). Similar results were observed at 48 h post-surgery (Fig. 7b, *P* < 0.05).

### miR-129-5p mimic prevented inflammation-induced damage to the BSCB after IR

As shown in Fig. 7c, almost no EB extravasation in the Sham group was visualized as red fluorescence. IR induced a robust increase in red fluorescence, especially in the gray matter, at 48 h post-surgery [2, 21]. Intrathecal injection of mimic-129 significantly decreased the degree and intensity of fluorescence and restricted it to the center of the gray matter, whereas injection of rHMGB1 or Poly(I:C) synergistically exacerbated BSCB leakage, as shown by the widespread distribution of red fluorescence throughout the spinal cord. There were no detectable differences between mice in the IR and con-129 groups (*P* > 0.05). Quantification of the EB content and the fluorescence intensity confirmed the above results (Fig. 7d, e, *P* < 0.05).

The increase in the spinal water content was likely attributable to BSCB leakage. The water content also showed similar trends to the EB extravasation results (Fig. 7f, *P* < 0.05).

## Discussion

Various miRs that are widely distributed in the CNS have been shown to play essential roles in modulating the pathogenesis of IR injury, thereby contributing to disease development and outcomes [10, 12]. In this study, we provided evidence for the first time that elevated miR-129-5p expression exerted neuroprotective effects in a mouse model of spinal cord IR.

miR-129-5p is known to regulate malignant tumor progression and metastasis [13]. Recently, emerging evidence has shown that miR-129-5p is dysregulated during trauma and degenerative and autoimmune diseases and thus has special significance for the maintenance of neuronal function and macrophage/monocyte migration under these pathological conditions [10, 14, 22]. Transfection of human osteoblast-like cells with an miR-129-3p mimic inhibits in vitro monocyte migration by targeting the human IL-17 gene, which is one of the major mechanisms underlying the pathogenesis of rheumatoid arthritis [22]. A study by Sepramaniam et al. suggested that among the various biomarkers identified, miR-129-5p was a significantly altered miR in stroke patients [23]. These findings indicate that miR-129-5p may be involved in regulating neuroinflammatory responses after injury. In this study, we demonstrated that miR-129-5p was downregulated in a temporal fashion and reached its lowest levels at both 12 and 48 h (Fig. 2), which was consistent with the results of our previous studies demonstrating the bimodal stages of IR-induced neuroinflammation and suggesting potential miRNA-based treatment strategies [21, 24].

We previously showed that some miRs are associated with TLR signaling pathways in IR [2]. Each miR can affect multiple target mRNAs, and each target mRNA can also be regulated by several miRs [1, 2, 20]. We used TargetScan to identify the targets of miR-129-5p by specifying a continuous “seed match” of more than six base pairs and a potential link with TLR signaling (e.g., adaptor, interacting proteins, and DAMPs). In a rat model of intracerebral hemorrhage, miR-129-5p has been shown to be associated with the inhibition of revascularization and angiogenesis by suppressing HMGB1 and RAGE signaling [14]. Thus, HMGB1 was selected as a potential target of miR-129-5p. The temporal expression patterns of HMGB1 and miR-129-5p preliminarily indicated a negative correlation between these two factors. This interaction was confirmed using a luciferase assay; a reduction in luciferase activity was observed in cells co-transfected with a vector containing the WT 3′UTR of HMGB1 and mimic-129 (Fig. 3). Furthermore, to account for the in vivo complexity and crosstalk, the above effects were also evaluated in a mouse model by pretreating the mice with mimic-129 and con-129 [2, 22]. Intrathecal injection of mimic-129 markedly reduced HMGB1 mRNA and protein expression, whereas these changes were not observed after treatment with con-129 (Fig. 5). Collectively, these data suggest that miR-129-5p functions as a negative modulator of HMGB1 in spinal cord IR.

HMGB1 is a highly conserved DNA-binding protein that functions as an intracellular regulator of nucleosome formation and gene transcription [25]. Recently, extracellular HMGB1 has been shown to be a potent proinflammatory factor that can lead to cytokine release and tissue damage [7, 9, 25]. Previous studies have demonstrated that HMGB1-mediated inflammation is a major mechanism in IR-induced brain damage [26]. Motor neurons are the most vulnerable to IR, and thus IR injury is usually associated with motor function impairments [21]. Using electron microscopy, Chen et al. demonstrated that translocation of HMGB1 from the nucleus to the cytoplasm in spinal neurons occurred within a few hours after trauma [7]. Additionally, the BSCB is critical for maintaining spinal homeostasis [4, 5]. Therefore, the role of HMGB1 in inflammatory responses against neurons and BSCB is most aptly represented by its specific cellular distribution. Spinal microglia, which are a type of innate immune cell, function as macrophages and are capable of phagocytosis immediately after IR [4]. Consistent with the study by Chen et al. [7], the immunofluorescence images in Fig. 4 show that overexpressed HMGB1 is mainly distributed in the cytoplasm of neurons and microglia. Additionally, the effects of miR-129-5p were measured at 48 h post-surgery when the maximal immunoreactivity against HMGB1 was observed. Compared with the IR group, the mimic-129 group had a markedly reduced number of double-labeled neurons and microglia, but these cells were relatively unchanged in the con-129 group, confirming that HMGB1 was released from stimulated neurons and activated microglia after IR (Fig. 6). Consistently, obvious improvement in hind-limb motor function and preservation of BSCB integrity were observed in the mimic-129 group, as demonstrated by elevated Basso Mouse Scale scores and decreased EB extravasation and water content (Fig. 7).

TLR3 is a unique member of the TLR family that is located in endosomal compartments or on the cell surface [27]. TLR3 usually recognizes pathogen-derived nucleic acids and recruits an adaptor protein to induce interferon-ß release [17, 28]. Recently, several studies investigated the essential roles of TLR3 activation in the amplification of inflammatory responses and cytokine release in response to HMGB1 [17, 29, 30]. Consistently, the increased immunoreactivity of HMGB1 in the cytoplasm coincided with the fluorescence signal representing TLR3 on the cell surface (Fig. 5). These increases in the translocation of TLR3 to the cell surface expose TLR3 to stimuli produced by injured cells [31]. In this study, intrathecal injection of rHMGB1 and Poly(I:C) both induced comparably higher levels of HMGB1 and TLR3 expression and led to robust secretion of IL-1β and TNF-α and BSCB leakage compared to the levels detected in the untreated IR group. These results are consistent with those of an in vivo study on alcohol-sensitized neuroinflammation [16]. In that study, sequential treatment with ethanol and Poly(I:C) facilitated the effects of Poly(I:C) on the induction of TLR3 and HMGB1 expression [16]. In another study, delayed release of IL-1β and TNF-α was observed in monocytes cultured with HMGB1 compared with monocytes directly treated with the TLR4 agonist lipopolysaccharide [7]. These findings indicate that HMGB1 may be a trigger for TLR3 activation. Early amplification of inflammatory responses can directly activate downstream signals without requiring HMGB1. Nevertheless, inhibition of HMGB1 with mimic-129 downregulated the expression of TLR3 and downstream cytokines, confirming that HMGB1 was required for TLR3 activation. In contrast, in a study by Yamagata et al., HMGB1 was not upregulated in astrocytes in response to stroke [32]. This difference might be attributable to the lack of effect of HMGB1 on intracellular NF-κB localization in cultured astrocytes because HMGB1 was shown to decrease the translocation of NF-κB to the nucleus during brain edema formation [33]. In addition to HMGB1, some indirect factors may also be involved in the IR injury process. Given the complexity and crosstalk among glial cells and neurons, further in vitro BSCB models need to be constructed to elucidate the underlying mechanisms and uncover new treatment strategies.

## Conclusions

In conclusion, this study explored the roles of miR-129-5p and its targets in spinal cord IR. Intrathecal pretreatment with mimic-129 protected neurons and the BSCB against inflammatory responses by downregulating HMGB1 expression and inhibiting TLR3-cytokine pathway activation. These results suggest the potential use of miRNA-based strategies for IR.

## Abbreviations

ANOVA: One-way analysis of variance
BSCB: Blood-spinal cord barrier
DAMPs: Damage-associated molecular pattern molecules
EB: Evans blue
HMGB1: High-mobility group box-1
IL: Interleukin
IR: Ischemia-reperfusion
miRs: MicroRNAs
MT: Mutant
NF-κB: Nuclear factor-kappa B
Poly(I:C): Polyinosinic-polycytidylic acid
RAGE: Receptor for advanced glycation end products
rHMGB1: Recombinant HMGB1
SD: Standard deviation
TLR: Toll-like receptor
TNF: Tumor necrosis factor
UTR: Untranslated region
WT: Wild-type

## References

[1] He F, Shi E, Yan L, Li J, Jiang X (2015). Inhibition of micro-ribonucleic acid-320 attenuates neurologic injuries after spinal cord ischemia. J Thorac Cardiovasc Surg.

[2] Li XQ, Lv HW, Tan WF, Wang ZL, Fang B, Ma H (2015). MiR-27a ameliorates inflammatory damage to the blood-spinal cord barrier after spinal cord ischemia-reperfusion injury in rats by down-regulating TICAM-2 of the TLR4 signaling pathway. J Neuroinflammation.

[3] Fang B, Li XQ, Bao NR, Tan WF, Chen FS, Pi XL (2016). Role of autophagy in the bimodal stage after spinal cord ischemia reperfusion injury in rats. Neuroscience.

[4] Bell MT, Puskas F, Agoston VA, Cleveland JC, Freeman KA, Gamboni F (2013). Toll-like receptor 4-dependent microglial activation mediates spinal cord ischemia-reperfusion injury. Circulation.

[5] Li XQ, Wang J, Fang B, Tan WF, Ma H (2014). Intrathecal antagonism of microglial TLR4 reduces inflammatory damage to blood-spinal cord barrier following ischemia/reperfusion injury in rats. Mol Brain.

[6] Zhao G, Fu C, Wang L, Zhu L, Yan Y, Xiang Y (2017). Down-regulation of nuclear HMGB1 reduces ischemia-induced HMGB1 translocation and release and protects against liver ischemia-reperfusion injury. Sci Rep.

[7] Chen KB, Uchida K, Nakajima H, Yayama T, Hirai T, Rodriguez Guerrero A (2011). High-mobility group box-1 and its receptors contribute to proinflammatory response in the acute phase of spinal cord injury in rats. Spine (Phila Pa 1976).

[8] Gu JJ, Chen JB, Zhang JH, Zhang H, Wang SS (2016). Recombinant human soluble thrombomodulin protects against brain injury in a CVST rat model, via downregulation of the HMGB1-RAGE axis. Mol Med Rep.

[9] Das N, Dewan V, Grace PM, Gunn RJ, Tamura R, Tzarum N (2016). HMGB1 activates proinflammatory signaling via TLR5 leading to allodynia. Cell Rep.

[10] Liu AH, Wu YT, Wang YP (2017). MicroRNA-129-5p inhibits the development of autoimmune encephalomyelitis-related epilepsy by targeting HMGB1 through the TLR4/NF-kB signaling pathway. Brain Res Bull.

[11] Zhai F, Zhang X, Guan Y, Yang X, Li Y, Song G (2015). Expression profiles of microRNAs after focal cerebral ischemia/reperfusion injury in rats. Neural Regen Res.

[12] Balsam LB. Spinal cordischemia-reperfusion injury: MicroRNAs and mitophagy at a crossroads. J Thorac Cardiovasc Surg. 2017; doi: 10.1016/j.jtcvs.2017.06.010. 10.1016/j.jtcvs.2017.06.010 28673704

[13] Kouhkan F, Mobarra N, Soufi-Zomorrod M, Keramati F, Hosseini Rad SM, Fathi-Roudsari M (2016). MicroRNA-129-1 acts as tumour suppressor and induces cell cycle arrest of GBM cancer cells through targeting IGF2BP3 and MAPK. J Med Genet.

[14] Ma XL, Li SY, Shang F (2017). Effect of microRNA-129-5p targeting HMGB1-RAGE signaling pathway on revascularization in a collagenase-induced intracerebral hemorrhage rat model. Biomed Pharmacother.

[15] Li C, Peng S, Liu X, Han C, Wang X, Jin T (2017). Glycyrrhizin, a direct HMGB1 antagonist, ameliorates inflammatory infiltration in a model of autoimmune thyroiditis via inhibition of TLR2-HMGB1 signaling. Thyroid.

[16] Qin L, Crews FT (2012). Chronic ethanol increases systemic TLR3 agonist-induced neuroinflammation and neurodegeneration. J Neuroinflammation.

[17] Yanai H, Ban T, Wang Z, Choi MK, Kawamura T, Negishi H (2009). HMGB proteins function as universal sentinels for nucleic-acid-mediated innate immune responses. Nature.

[18] Alexopoulou L, Holt AC, Medzhitov R, Flavell RA (2001). Recognition of double-stranded RNA and activation of NF-kappaB by Toll-like receptor 3. Nature.

[19] Zhang H, Verkman AS (2014). Longitudinally extensive NMO spinal cord pathology produced by passive transfer of NMO-IgG in mice lacking complement inhibitor CD59. J Autoimmun.

[20] Luo J, Chen J, He L (2015). mir-129-5p attenuates irradiation-induced autophagy and decreases radioresistance of breast cancer cells by targeting HMGB1. Med Sci Monit.

[21] Li XQ, Lv HW, Tan WF, Fang B, Wang H, Ma H (2014). Role of the TLR4 pathway in blood-spinal cord barrier dysfunction during the bimodal stage after ischemia/reperfusion injury in rats. J Neuroinflammation.

[22] Tsai CH, Liu SC, Wang YH, Su CM, Huang CC, Hsu CJ (2017). Osteopontin inhibition of miR-129-3p enhances IL-17 expression and monocyte migration in rheumatoid arthritis. Biochim Biophys Acta.

[23] Sepramaniam S, Tan JR, Tan KS, DeSilva DA, Tavintharan S, Woon FP (2014). Circulating microRNAs as biomarkers of acute stroke. Int J Mol Sci.

[24] Li XQ, Fang B, Tan WF, Wang ZL, Sun XJ, Zhang ZZ (2016). miR-320a affects spinal cord edema through negatively regulating aquaporin-1 of blood–spinal cord barrier during bimodal stage after ischemia reperfusion injury in rats. BMC Neurosci.

[25] Stevens NE, Chapman MJ, Fraser CK, Kuchel TR, Hayball JD, Diener KR (2017). Therapeutic targeting of HMGB1 during experimental sepsis modulates the inflammatory cytokine profile to one associated with improved clinical outcomes. Sci Rep.

[26] Zheng C, Liu C, Wang W, Tang G, Dong L, Zhou J (2016). Ethanol extracts from Portulaca oleracea L. attenuated ischemia/reperfusion induced rat neural injury through inhibition of HMGB1 induced inflammation. Am J Transl Res.

[27] Jiang W, Sun R, Wei H, Tian Z (2005). Toll-like receptor 3 ligand attenuates LPS-induced liver injury by down-regulation of toll-like receptor 4 expression on macrophages. Proc Natl Acad Sci U S A.

[28] Lai R, Gu M, Jiang W, Lin W, Xu P, Liu Z (2017). Raf kinase inhibitor protein preferentially promotes TLR3-triggered signaling and inflammation. J Immunol.

[29] Garg AD, Nowis D, Golab J, Vandenabeele P, Krysko DV, Agostinis P (1805). Immunogenic cell death, DAMPs and anticancer therapeutics: an emerging amalgamation. Biochim Biophys Acta.

[30] Li H, Li Q, Guo T, He W, Dong C, Wang Y (2017). LncRNA CRNDE triggers inflammation through the TLR3-NF-κB-cytokine signaling pathway. Tumour Biol.

[31] Suresh MV, Thomas B, Machado-Aranda D, Dolgachev VA, Kumar Ramakrishnan S, Talarico N (2016). Double-stranded RNA interacts with toll-like receptor 3 in driving the acute inflammatory response following lung contusion. Crit Care Med.

[32] Yamagata K, Sone N, Suguyama S, Nabika T (2016). Different effects of arginine vasopressin on high-mobility group box 1 expression in astrocytes isolated from stroke-prone spontaneously hypertensive rats and congenic SHRpch1_18 rats. Int J Exp Pathol.

[33] Ohnishi M, Monda A, Takemoto R, Fujimoto Y, Sugitani M, Iwamura T (2014). High-mobility group box 1 up-regulates aquaporin 4 expression via microglia-astrocyte interaction. Neurochem Int.

