# Correction to: Elevated microRNA-129-5p level ameliorates neuroinflammation and blood-spinal cord barrier damage after ischemia–reperfusion by inhibiting HMGB1 and the TLR3-cytokine pathway

**DOI:** 10.1186/s12974-021-02345-2

**Published:** 2021-12-28

**Authors:** Xiao-Qian Li, Feng-Shou Chen, Wen-Fei Tan, Bo Fang, Zai-Li Zhang, Hong Ma

**Affiliations:** Department of Anesthesiology, First Affiliated Hospital, China Medical University, Shenyang, 110001 Liaoning China

## Correction to: J Neuroinflamm (2017) 14:205 https://doi.org/10.1186/s12974-017-0977-4

The original version of the article [1] unfortunately contained a mistake. The error occurred in representative images of double immunofluorescence of Fig. 6.

**Fig. 6:**
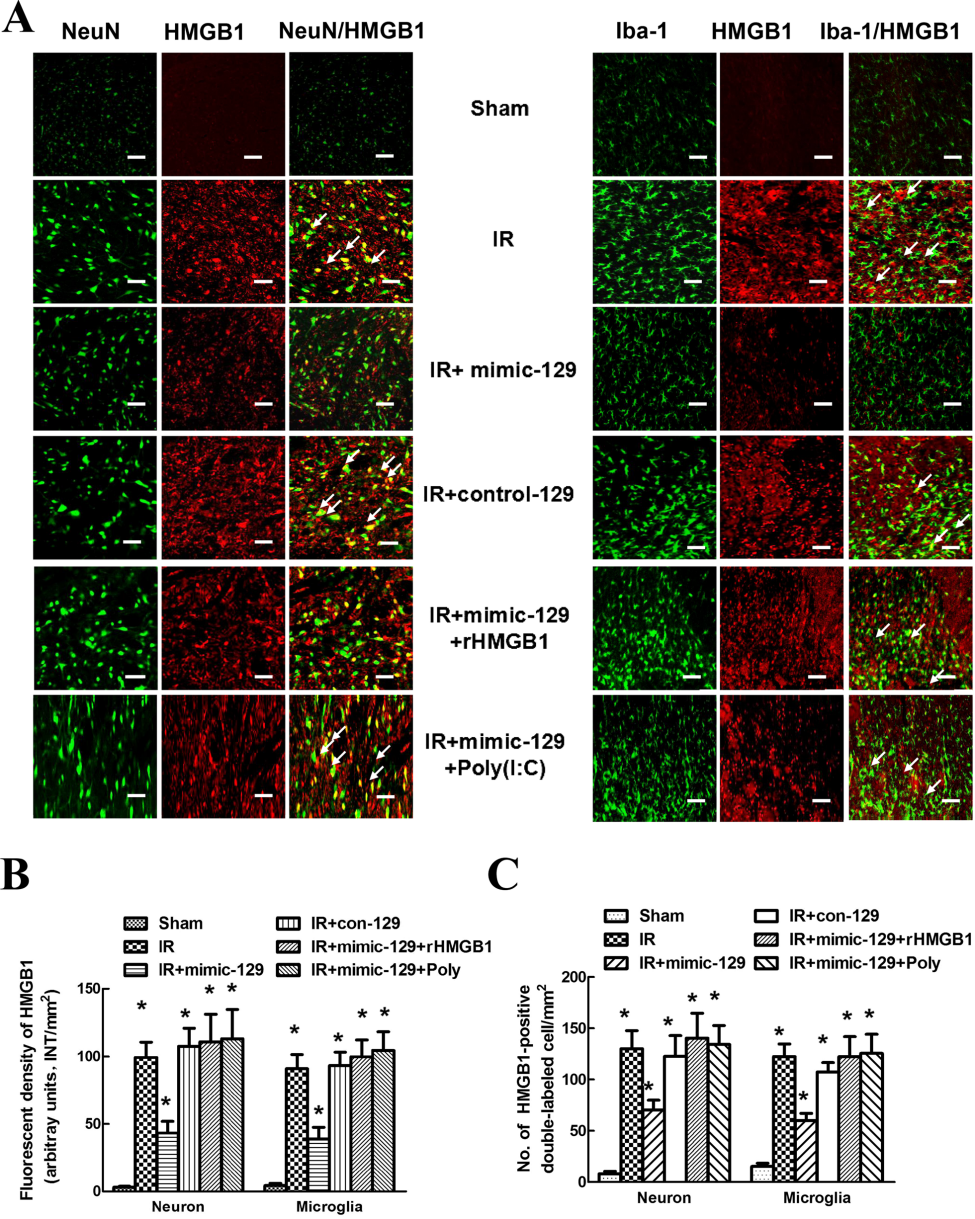
Effects of the miR-129-5p mimic and mimic control on HMGB1 expression in specific cell types of the spinal cord after IR. **a** Representative photomicrographs showing the localization of the fluorescence signals for HMGB1 in neurons and microglia at 48 h after IR. Arrows indicate co-localization. Scale bars = 50 μm. **b** Quantification of HMGB1 signals was performed based on the average of three independent images. **c** Quantification of HMGB1-positive neurons and microglia in the spinal cords at 48 h after IR. Data are expressed as the mean ± SD. *P < 0.05 versus the Sham group. ^#^*P* < 0.05 versus the IR group

The other data and the conclusion in the publication are real and reliable.

It has been corrected in this correction.

The correct version of Fig. 6 is given in this Correction article.
